# Cysteinyl-Leukotriene Receptors and Cellular Signals

**DOI:** 10.1100/tsw.2007.185

**Published:** 2007-09-01

**Authors:** G. Enrico Rovati, Valérie Capra

**Affiliations:** Laboratory of Molecular Pharmacology, Department of Pharmacological Sciences, University of Milan, Via Balzaretti 9, 20133 Milan, Italy

**Keywords:** CysLT_1_, CysLT_2_, GPR17, signaling pathway

## Abstract

Cysteinyl-leukotrienes (cysteinyl-LTs) exert a range of proinflammatory effects, such as constriction of airways and vascular smooth muscle, increase of endothelial cell permeability leading to plasma exudation and edema, and enhanced mucus secretion. They have proved to be important mediators in asthma, allergic rhinitis, and other inflammatory conditions, including cardiovascular diseases, cancer, atopic dermatitis, and urticaria. The classification into subtypes of the cysteinyl-LT receptors (CysLTRs) was based initially on binding and functional data, obtained using the natural agonists and a wide range of antagonists. CysLTRs have proved remarkably resistant to cloning. However, in 1999 and 2000, the CysLT_1_R and CysLT_2_R were successfully cloned and both shown to be members of the G-protein coupled receptors (GPCRs) superfamily. Molecular cloning has confirmed most of the previous pharmacological characterization and identified distinct expression patterns only partially overlapping. Recombinant CysLTRs couple to the G_q/11_ pathway that modulates inositol phospholipids hydrolysis and calcium mobilization, whereas in native systems, they often activate a pertussis toxin-insensitive G_i/o_-protein, or are coupled promiscuously to both G-proteins. Interestingly, recent data provide evidence for the existence of an additional receptor subtype that seems to respond to both cysteinyl-LTs and uracil nucleosides, and of an intracellular pool of CysLTRs that may have roles different from those of plasma membrane receptors. Finally, a cross-talk between the cysteinyl-LT and the purine systems is being delineated. This review will summarize recent data derived from studies on the molecular and cellular pharmacology of CysLTRs.

### CYSTEINYL-LT/CYSLT RECEPTOR SYSTEM: THE PAST

Cysteinyl-leukotrienes (cysteinyl-LTs) are potent lipid mediators synthesized from arachidonic acid in response to different immune and inflammatory stimuli[1,2]. They have recognized roles in respiratory diseases, such as asthma and allergic rhinitis, but have been implicated in other inflammatory conditions, including cancer, cardiovascular, gastrointestinal, skin, and immune disorders[3]. Early pharmacological studies have provided evidence that these lipid mediators exert their actions through specific cellular targets, namely, receptor proteins, while subsequent experimental evidence strongly suggested that these are G-protein coupled receptors (GPCRs) (for a complete review, see [4,5,6,7,8]).

Over the last 20 years, a large number of selective antagonists for cysteinyl-LT receptors (CysLTRs) have been developed[9]. The use of these antagonists enabled an initial pharmacological classification of CysLTRs in two broad subgroups: those that were blocked by these antagonists ($CysLT_1$)[10,11,12] and those that were resistant to blockade ($CysLT_2$). Currently, only BAY u9773 displays antagonist actions at both receptors, unfortunately with poor potency and selectivity, especially in human tissues[13,14].

### CYSLTR CLONING: THE BREAKTHROUGH

CysLTRs escaped gene cloning with conventional approaches up to 1999, when two separate groups[15,16] cloned the first CysLTR using a cognate ligands strategy for fishing orphan GPCRs. Soon, thereafter, almost simultaneously, came the $CysLT_2R$ cloning by three different groups[17,18,19]. Previous biochemical and pharmacological findings were essentially confirmed by the sequence analysis demonstrating their belonging to the GPCR group of receptors organized in the characteristic seven serpentine topology. They belong to the rhodopsin family of the GPCR gene superfamily and, in particular, to the purine receptor cluster (within the δ group) of phylogenetically related receptors, which includes, besides a number of orphans, receptors that respond to purinergic or pyrimidinergic nucleotides (P2Ys), proteases (F2Rs), and platelet activating factor (PAF) (PAFR)[20,21]. Unlike the monoamine or neuropeptide receptors, the receptors belonging to the purine cluster have no clear homologues in invertebrates, suggesting a relatively recent evolutionary origin[22,23]. Both receptors are glycosylated protein, sharing only 38% amino acid (aa) identity, with very low homology in the extreme carboxyl termini.

The gene/chromosome location is also known. Human $CysLT_1R$ and $CysLT_2R$ are located on the long arms of chromosomes X (Xq13-Xq21)[15] and 13 (13q14)[17,18], respectively.

The basic characteristics of the human $CysLT_1R$ and $CysLT_2R$ are summarized in Table 1.

**TABLE 1**
**Schematic Characteristics of Cloned Human CysLTRs**

| | $CysLT_1R$ | $CysLT_2R$ | GPR17($CysLT_3R$) |
|---|---|---|---|
| Gene symbol* | CYSLTR1 | CYSLTR2 | GPR17 |
| Genomic location | Xq13-Xq21 | 13q14.2 | 2q21 |
| Accession number (GenBank) | AF119711 | AB038269 | NM005291 |
| Protein size | 337 aa | 346 aa | 339 aa |
| Pharmacological profile | $LTD_4 > LTC_4 >> LTE_4$ | $LTD_4 = LTC_4 >> LTE_4$ | $LTC_4 > LTD_4$ |
| Primary coupling** | Gq/11 | Gq/11 | Gi |
| Primary expression | Peripheral blood leukocytes, spleen, smooth muscle (lung, intestine) | Peripheral blood leukocytes, spleen, adrenal medulla, heart, brain | Brain, heart, kidney |

* Symbol approved by the HUGO Gene Nomenclature Committee (HGNC) (http://www.gene.ucl.ac.uk/nomenclature/)

** See text for further information on the coupling of native CysLTRs.

### $CysLT_1R$

#### *Molecular Biology*

The open reading frame for the human $CysLT_1R$ encodes a protein of 337 aa with a calculated molecular mass of 38 kDa[15,16]. It was observed to migrate at a molecular weight of 30–42 kDa (depending on experimental conditions) as a monomeric form[24,25,26,27,28,29], although oligomers were often observed (see “Oligomerization”).

The genomic structure and transcriptional regulation have been studied, and it was revealed that the human $CysLT_1R$ contains five exons that are variably spliced and a single promoter region with multiple transcription start sites, and that the entire open reading frame is located in exon 5[30]. Multiple splice variants of $CysLT_1R$ exist and the transcript expression patterns differ from tissues and cell types[30,31].

Human $CysLT_1R$ possesses four potential N-glycosylation sites besides many potential protein kinase A and C phosphorylation sites, mostly located in the third intracellular loop and carboxyl terminal[15].

Human $CysLT_1R$ has the highest homology (32% aa identity) with the purinoceptor P2Y1 and the receptor for PAF, whereas it shares a lower homology (28% aa identity) with the other subclasses of leukotriene receptors, the BLT receptors[15].

#### *Binding Studies*

Binding studies with $[^3H]LTD_4$ on membranes from host cells transiently expressing the receptor identified a high-affinity binding with a $K_d$ of 0.3–9 n*M*[15,16] compatible with the values reported for other human tissues and cells[32,33,34,35]. The affinity of $LTC_4$ is about 350-fold lower than that of $LTD_4$[15], in agreement with data obtained from human lung membranes[34]. Furthermore, $[^3H]LTD_4$ binding is potently and competitively inhibited by members of structurally distinct classes of $CysLT_1R$ antagonists, such as montelukast, zafirlukast, and pranlukast ($IC_{50}$ in the range of 1.8–4.9 n*M*) and to a lesser extent by pobilukast ($IC_{50}$ = 30 n*M*)[16].

#### *Pharmacological Characterization and Signal Transduction in Recombinant Systems*

$LTD_4$ displayed a potency higher than that of $LTC_4$ and $LTE_4$, in cells transfected with the recombinant $CysLT_1R$. Indeed, characterization of $CysLT_1R$ activation on cRNA-injected melanophores from *Xenopus laevis* demonstrated that $LTD_4$ is the most potent cysteinyl-LT agonist with an $EC_{50}$ of 0.4 n*M*, whereas that of $LTC_4$ is 21 n*M*[15]. The same rank order of potency was observed in other functional assays in *X. laevis* oocytes or in $Ca^{2+}$ mobilization assay in COS-7[15,16], HEK-293, and CHO[16] (see also [3]). $LTE_4$ is the less potent leukotriene agonist acting as a partial agonist[16], in agreement with data previously obtained for other human tissues[13,36]. As expected, the $LTD_4$ functional response is potently inhibited by the selective $CysLT_1R$ antagonists MK571[15], zafirlukast, pranlukast, montelukast, and pobilukast[16].

Interestingly, in the recombinant systems, $CysLT_1R$ appeared very weakly, if not at all, coupled to a pertussis toxin (PTX)-sensitive G-protein[15,16], in agreement with previous results obtained in animal tissues[37]. However, it is known that the use of recombinant systems might produce results that depend on cell type, transducer, or effector availability, especially when dealing with GPCRs[38]. Indeed, many research groups observed coupling also to $G_{i/o}$ family in human cells[36,39,40,41] (see below).

#### *Regulation of Functionality*

Agonist-induced internalization of recombinant human $CysLT_1R$ has been reported to be GRK/arrestin independent and significantly PKC dependent, particularly in COS-1 transfected cells[42]. At variance with these data, $CysLT_1R$ homologous desensitization in the human macrophage-like cell line U937 most likely depends on GRK2 activation[43]. Interestingly, extracellular nucleotide-induced $CysLT_1R$ heterologous desensitization, which was indeed dependent on PKC, did not cause receptor internalization and induced a very fast recovery of $CysLT_1R$ functionality with respect to agonist-induced homologous desensitization and trafficking[43] (see also “Cross-Talk with Other GCPRs”).

#### *Distribution in Human Tissues*

The pathophysiological role of cysteinyl-LTs in asthma is well documented[44,45,46,47], and results obtained from localization studies are coherent with the antibronchoconstrictive and anti-inflammatory activities of $CysLT_1R$ antagonists[15,24]. Findings from *in situ* hybridization analysis indicates $CysLT_1R$ mRNA expression in the smooth muscle cells (SMCs) at all levels of the respiratory tree as well as in interstitial lung macrophages, and little expression in the epithelial cells[15]. $CysLT_1R$ has been localized at gene and protein level, in infiltrating and structural cells of human nasal mucosa from normal subjects[48], and patients with asthma/allergic rhinitis[49] or aspirin-sensitive/aspirin-tolerant chronic rhinosinusitis[50].

Immunohistochemical analysis on peripheral blood leukocytes (PBLs) showed the presence of $CysLT_1R$ in a series of cells of particular relevance to asthma and atopy, such as monocytes/macrophages, eosinophils, pregranulocytic $CD34^+$ cells, neutrophils, and in subsets of B lymphocytes, but not T lymphocytes[24]. Gauvreau and colleagues[51] documented that basophils, which accumulate in the airways of subjects with atopic asthma[52], express variable levels of functional $CysLT_1R$. Furthermore, $CysLT_1R$ expression was demonstrated in human mast cells (MCs) from normal[27,53] and asthmatic subjects[54].

In the gastrointestinal system, $CysLT_1R$ expression has been documented in small intestines and colon[15,16], in colorectal carcinomas, and in colon cancer cells[28] (for details, see article by Massoumi and Sjölander[134]). The expression of a functional $CysLT_1R$ has also been reported in human saphenous veins, where it mediates contractile effects of cysteinyl-LTs[55] (for more detailed information on LT receptors in vascular SMCs, see article by Bäck[135]). Little expression has been initially reported in brain[15,16], but then documented in human brains with traumatic injury or tumors[56].

For a more detailed description of $CysLT_1R$ distribution and functional significance, see Capra et al.[3].

### $CysLT_2R$

#### *Molecular Biology*

Gene cloning and characterization studies of human $CysLT_2R$ were first reported by Heise and coworkers[17], and then confirmed and expanded by two other groups[18,19]. The open reading frame of human $CysLT_2R$ encodes a protein of 346 aa, which appears to migrate at a molecular weight of 58 kDa in basophil lysates[51].

Human $CysLT_2R$ possesses four potential N-glycosylation sites, three of which are in the extracellular N-tail, besides many potential protein kinase A and C phosphorylation sites mostly located in the third intracellular loop and carboxyl terminal[17].

The genomic organization of the human $CysLT_2R$ has not yet been published.

#### Binding Studies

Data analysis of saturation binding experiments with [$^3$H]$LTD_4$ in COS-7 cell membranes transiently expressing $CysLT_2R$ revealed the presence of high- and low-affinity sites ($K_{d1}$ = 0.4 n*M* – $K_{d2}$ = 51 n*M*)[17]. Takasaki and colleagues, who used [$^3$H]$LTC_4$ in the presence of s-decylglutathione[18] to disguise the binding of $LTC_4$ to its other nonreceptor sites[34], observed affinity in the nanomolar range ($K_d$ = 3 n*M*). Furthermore, all the classical $CysLT_1R$ antagonists were demonstrated to be inactive in competition assays, whereas BAY u9773 was a full competitor[17,19].

### *Pharmacological Characterization and Signal Transduction in Recombinant Systems*

Characterization of the cloned receptor has mostly confirmed the data previously reported in the literature[13]. Functional activation by aequorin assay in HEK293T cells transiently expressing the receptor demonstrated that $LTD_4$ and $LTC_4$ are equipotent agonists, whereas $LTE_4$, again, behaves as a partial agonist[17]. Similar findings were obtained in other functional activation assays in *X. laevis* oocytes[17] or in $Ca^{2+}$ mobilization assay in HEK293T cells[18] (see also [3]). As expected, the response of this receptor subtype was characterized by the lack of sensitivity to the classical $CysLT_1R$ antagonists MK571, montelukast, zafirlukast, and pranlukast[17]. BAY u9773, which displayed the expected antagonist activity, intriguingly acted in a noncompetitive manner against $LTC_4$[17], despite behaving as a full competitor in binding assay with [$^3$H]$LTD_4$ (see above)[17,19]. Furthermore, Nothacker and colleagues[19] highlighted the BAY u9773 role as a subtype selective agonist to the recombinant $CysLT_2R$, in agreement with the previous findings of Labat and coworkers on human bronchus and pulmonary veins[13].

Functional assay on *X. laevis* oocytes indicated that, at least in this system, $CysLT_2R$ is not coupled to a PTX-sensitive G-protein[17]. Again, the same is also true in human umbilical endothelial cells (HUVECs) (G.E. Rovati and A. Sala, unpublished observations), but not in human MCs[57].

No data are yet available on the regulation of $CysLT_2R$ functionality.

### *Distribution in Human Tissues*

Localization studies have been performed with various techniques and have identified a distinctive expression pattern for human $CysLT_2R$, despite some overlapping with $CysLT_1R$. Indeed, expression in heart, brain, and adrenals appears to be peculiar to $CysLT_2R$.

$CysLT_2R$ mRNA is highly expressed in the entire heart, as revealed by Northern analysis[17,18,19]. Further characterization by *in situ* hybridization indicated a particular concentration in Purkinje fiber cells[17], myocytes and fibroblasts derived from atrium and ventricle, coronary artery–derived SMCs, and lack of expression in endothelial cells (ECs)[58]. Human saphenous veins express $CysLT_2R$ that are not implicated in contraction and for which a functional role remains to be determined[55]. Several authors indicate that HUVECs almost exclusively express $CysLT_2R$[59,60,61]. Immunohistochemical analysis of brain tissues from patients undergoing brain surgery indicates that $CysLT_2R$ is expressed in the SMCs, but not in the ECs, of arteries and veins of normal area[62] (for more detailed information on LT receptors in vessels and endothelium[135]).

$CysLT_2R$ mRNA is highly expressed in several regions of the brain, with particular concentration in hypothalamus, thalamus, putamen, pituitary, and medulla[17]. Its expression has been reported in the granulocytes of the brain parenchyma, and in neuron- and glial-appearing cells in either the late stages of traumatic injury or in the area surrounding the tumors, and in the ECs of microvessels that regenerate after traumatic brain injury[62].

The adrenal gland may represent a novel tissue for future studies on cysteinyl-LT functions and the $CysLT_2R$ role in modulating endocrine system because a very good level of expression was detected, particularly in medullary pheochromocytes[15]. In the immune system, moderate expression of $CysLT_2$

mRNA was seen in spleen, lymph nodes, and PBLs, with very strong expression in eosinophils[17,59], suggesting unidentified roles for this receptor in these cells. Mellor and colleagues[57] reported that human MCs also constitutively express the type-2 receptor for cysteinyl-LTs, and $CysLT_2R$ expression was reported in basophils[51]. No expression was found in either undifferentiated or differentiated promyelocytic HL-60 and promonocytic U937 cells, which are known to express high levels of $CysLT_1R$[19,43].

In human lung, the $CysLT_2$ mRNA signal was very high in interstitial macrophages and weak in SMCs[17]. A549 cells, a human lung adenocarcinoma-derived line with alveolar epithelial cell properties, express mRNA for $CysLT_2R$, but not for $CysLT_1R$[63].

For a more detailed description of $CysLT_2R$ distribution and functional significance see Capra et al.[3].

### CELLULAR SIGNALING IN CONSTITUTIVE HUMAN SYSTEMS

#### $CysLT_1$ Functions and Cellular Signaling

In consideration of the widely known spasmogenic activities of cysteinyl-LTs[64], intracellular $Ca^{2+}$ mobilization and phosphatidylinositol (PI) metabolism were the obvious signal transduction systems to investigate. U937 cells, a promonocytic leukemia cell line known, on differentiation with dimethyl sulfoxide (DMSO), to express a high density of $CysLT_1R$ endogenously[65], is certainly the first human cell line in which cysteinyl-LT signal transduction mechanisms have been studied. The group of Crooke first demonstrated that in DMSO-differentiated U937 (dU937), $LTD_4$ is able to induce elevation of intracellular free $Ca^{2+}$ concentration ($[Ca^{2+}]_i$) and PI metabolism[66]. In the same cells, the same authors further showed that treatment with phorbol 12-myristate 13-acetate (PMA) blocked $LTD_4$-induced PI metabolism and $Ca^{2+}$ mobilization[67,68] and that, accordingly, the inhibitor of PKC staurosporine augmented $LTD_4$-induced PI metabolism and $[Ca^{2+}]_i$. These observations suggested for the first time that PKC might play a role in determining the responsiveness of $CysLT_1R$[69]. Indeed, heterologous desensitization of $CysLT_1R$ is mediated through activation of PKC in dU937 cells[70] (see also “Cross-Talk with Other GPCRs”). $[Ca^{2+}]_i$ elevation has also been demonstrated in THP-1, another monocytic leukemia cell line[40,71], and more recently in human MCs[53] and monocyte-derived macrophages[61]. Furthermore, in dU937 cells, $Ca^{2+}$ signaling in response to $LTD_4$ appears to be a prenylated protein-dependent phenomenon[35].

Specifically considering the crucial bronchoconstrictor activity of cysteinyl-LTs[72] and their role in asthma[3,45], a number of studies have focused on human airways. Early studies in human bronchial muscle preparations suggested that the CysLTR in this system might be linked with a receptor-operated calcium-entry mechanism[73]. Accordingly, in freshly isolated SMCs from human small bronchioles, $LTD_4$ caused a slow increase in $[Ca^{2+}]_i$, with a consequent rise of the activity of large conductance $Ca^{2+}$-dependent $K^+$ channels and the amplitude of depolarization-induced outward whole-cell current[74]. These data seem to suggest that $LTD_4$ causes constriction of these small bronchioles primarily by activating $Ca^{2+}$ entry via nonvoltage gated channels, possibly by a PC-PLC mediated pathway. However, $LTD_4$ contraction of SMCs from human bronchi was found to be partially $Ca^{2+}$ independent, involving both $Ca^{2+}$-dependent and -independent isoforms of PKC[75].

It was again in U937 cells that $CysLT_1R$ have been demonstrated for the first time to modulate $Ca^{2+}$ responses through at least two G-proteins, one PTX sensitive ($G_{i/o}$) and one insensitive ($G_{q/11}$)[35,36,68]. These data are in good agreement with the finding that $LTD_4$ activates distinct signaling pathways differently coupled to G-proteins also in THP-1 cells: a PTX-insensitive mitogen-activated protein kinase (MAPK) activation and a PTX-sensitive chemotactic response[40]. More recently, it has been recognized that $CysLT_1R$ activation induces MAPK phosphorylation through a $G_{i/o}$-protein in mesangial[76], dU937[29], airway SMCs[41], and MCs[77]. Finally, in bronchial SMCs, $CysLT_1R$-dependent actin reorganization is coupled with PTX-sensitive G-protein[78]. Thus, these data confirm $CysLT_1R$

promiscuity in G-protein coupling in constitutive systems, at variance with data obtained, so far, in recombinant systems.

In bronchial SMCs, $LTD_4$-induced actin reorganization through a $CysLT_1R$ is extremely dependent on Rho GTPases and tyrosine phosphorylation pathways[78]. These experiments have been corroborated by the fact that cysteinyl-LTs play an augmentative role in human airway smooth muscle (ASM) migration, and the use of a phosphatidylinositol-3 kinase (PI3K) inhibitor suggests that this pathway is a key signaling mechanism in the chemotactic migration of ASM cells in response to cysteinyl-LTs[79].

Despite that airway constriction has always been considered the main function of $CysLT_1R$ in the respiratory tree, $LTD_4$ also induces proliferation of human malignant hematopoietic cell lines[80], airway epithelial cells[81], airway SMCs[41,82,83,84,85], renal mesangial cells[76,86], eosinophil hematopoietic progenitor cells[87], and lung fibroblasts[88]. Many are the mechanisms through which GPCRs may induce cell proliferation. One of the possible pathways requires the phosphorylation of the extracellular-signal regulated kinase 1/2 (ERK1/2), sometimes through the transactivation of a growth factor receptor. A first report suggested that $LTD_4$ synergizes with the insulin grow factor (IGF) axis to induce airway SMC proliferation, demonstrating proteolysis of airway SMC-produced inhibitory IGF-binding proteins (IGFBP) by $LTD_4$-induced matrix metalloproteinase (MMP)-1[82]. In the same system, $LTD_4$ has been demonstrated to induce phosphorylation of apoptosis signal-regulating kinase 1 (ASK1)[85], an upstream kinase kinase of c-Jun-NH(2)-terminal kinase (JNK) and p38 MAPK, which in turn regulates transcription factor activator protein-1 (AP-1), an essential step for regulation of cell proliferation and differentiation. In line with these results, $LTD_4$-induced airway SMC proliferation was recently demonstrated to require transactivation of the epidermal growth factor (EGF) receptor through generation of reactive oxygen species and ERK1/2 phosphorylation[41]. Furthermore, human lung fibroblasts have been demonstrated to proliferate in response to the conditioned medium obtained from epithelial cells in which $LTC_4$ stimulates the production of transforming growth factor beta1 (TGF-$\beta$1) through a p38 MAPK activation mechanism[88]. As mentioned before, $LTD_4$ has been postulated to activate MAPK in THP-1 through a PKC-Raf-1–dependent pathway[40], whereas in dU937 cells ERK1/2 activation involves a RasGTP-dependent pathway, PLC, and $Ca^{2+}$-dependent tyrosine kinase(s)[29]. In renal mesangial cell, $LTD_4$-induced proliferation requires ERK and p38 activation, and is dependent on PI3K and PKC[76]. The same group also recognized that $LTD_4$ transactivates the platelet-derived growth factor (PDGF) receptor beta, a process associated with c-Src recruitment and Ras activation, and that c-Src activation was insensitive to PTX[86]. In human MCs, $LTD_4$ enhanced proliferation in a $CysLT_1$- and ERK-dependent manner, which required transactivation of c-kit[77]. To our knowledge, there is only one report suggesting that cysteinyl-LTs may, on the contrary, inhibit the growth of a human cell line, i.e., the mammary cancer cells MCF-7[89].

In some systems, $CysLT_1R$ activation can contribute to the propagation of the inflammatory reaction by the release of various mediators. In U937 cells, $LTD_4$ triggered a rapid release of arachidonic acid metabolites into the culture medium, which was suppressed by the $CysLT_1R$ antagonist SK&F 104353, by the topoisomerase I inhibitor camptothecin, and by staurosporine[90]. In ECs, CysLTR were linked to the formation of nitric oxide[91], which, however, is not completely blocked by $CysLT_1R$ antagonists[92]. In human MCs primed with IL-4, the $CysLT_1$-selective receptor antagonist MK571 did inhibit, besides $Ca^{2+}$ flux, production of IL-5, tumor necrosis factor (TNF)-alpha, and large quantities of macrophage inflammatory protein (MIP)-1beta[93]. Cysteinyl-LTs were demonstrated to induce the release of MCP-1 in human monocytes/macrophages[94], an event that may occur also in cooperation with IL-4 and is mediated through the $CysLT_1R$, because in IL-4–primed THP-1 cells, MCP-1 increase was effectively inhibited by the $CysLT_1R$-selective antagonist MK571 and only partially by the nonselective antagonist BAY u9773[30]. Very recently, it has been shown that $LTD_4$ could up-regulate the expression of IL-8 in monocytic cells via the $CysLT_1R$[95]. The same group further studied $CysLT_1R$ signaling in HEK293, stably expressing the receptor showing modulation of IL-8 production by $LTD_4$ and demonstrating of the involvement of the NF-κB and AP-1 pathways.

Interestingly, some authors found that several-day stimulation of basophils in culture with $LTD_4$ reduced the frequency of CD95 Fas receptor expression, an effect that, albeit modest, was reversed by the

$CysLT_1R$ antagonist zafirlukast[51], suggesting that the cysteinyl-LT/$CysLT_1R$ system might have a direct effect on basophil accumulation in allergic tissues.

Another interesting function that has been postulated for the cysteinyl-LT/$CysLT_1R$ system is the regulation of hematopoietic stem and progenitor cell mobilization and homing, as $LTD_4$-induced $CysLT_1R$ activation seems to up-regulate adhesion of $CD34^+$ progenitors to primary bone marrow ECs through the intervention of β1-integrins[96].

Finally, in human airway, $CysLT_1R$ activates PKC to modulate the $beta_2$ adrenoreceptor ($\beta_2AR$) activity, causing its desensitization and a reduced response to $\beta_2$ agonists *in vitro*[97] (see also "Cross-Talk with Other GPGRs"). This phenomenon, besides agonist-induced tolerance, might contribute to the progressive fading of $\beta_2$ agonist efficacy observed in asthmatics[98] and may open up new strategies for asthma treatment[99].

### $CysLT_2$ Function and Cellular Signaling

Detailed information about signal transduction systems involved in $CysLT_2R$ activation is still lacking and hampered by the absence of selective antagonists. Contrasting reports are present in the literature regarding $Ca^{2+}$ signaling in HUVECs. $LTC_4$ and $LTD_4$ were reported to induce a rapid rise of $[Ca^{2+}]_i$, which is inhibited by the receptor antagonist SKF 104,353[100]. In agreement with these findings, another group has identified $CysLT_1R$ expression in these cells[26]. However, soon thereafter, it was demonstrated that these cells almost exclusively express the $CysLT_2R$[60], which appears to be strongly up-regulated by IL-4[61] and responsible for the $Ca^{2+}$ mobilization and contraction evoked by cysteinyl-LTs[61] and BAY u9773[60].

As for the $CysLT_1R$, the $CysLT_2R$ also appears to be coupled to PTX-insensitive G-proteins in recombinant systems (see above). Again, while this is true in HUVECs, where the $Ca^{2+}$ mobilization elicited by $CysLT_2R$ activation is totally PTX insensitive (G.E. Rovati and A. Sala, unpublished observations), recently, Mellor and colleagues[57] demonstrated in human MCs that $CysLT_2R$ signaling was completely inhibited by PTX. This group reported that human MCs, besides $CysLT_1R$, express $CysLT_2R$ as well, and that the selective function of $CysLT_2R$ was evident based on uninhibited IL-8 secretion in IL-4–primed cells stimulated with cysteinyl-LTs or UDP by the presence of the selective $CysLT_1R$ antagonist MK571. IL-8 generation was evoked also by the $CysLT_2R$ partial agonist BAY u9773 and was inhibited by the selective p38 kinase inhibitor, SB203580[57].

In HUVECs, $LTD_4$-induced $CysLT_2R$ activation was found to up-regulate 37 early inducible genes, among which the most strongly induced were early growth response (EGR), nuclear receptor subfamily 4 group A transcription factors, E-selectin, CXC ligand 2, IL-8, a disintegrin-like and metalloprotease (reprolysin type) with thrombospondin type 1 motif 1 (ADAMTS1), Down syndrome critical region gene 1 (DSCR1), tissue factor (TF), and cyclooxygenase 2 (COX-2)[101].

Previous studies in ECs postulated that cysteinyl-LTs may be relevant to inflammation, hemostasis, thrombosis, and mechanisms of vascular injury, including atherosclerosis, as it was demonstrated that cysteinyl-LTs can trigger several other functional responses, such as synthesis of PAF, secretion of von Willebrand factor, and expression of P-selectin[102,103,104]. In light of recent findings, it is clear why the expression of endothelial P-selectin induced by either $LTC_4$ or $LTD_4$ was not blocked by pretreatment of HUVECs with selective $CysLT_1R$ antagonists[104]. Significance of cysteinyl-LT involvement in the pathogenesis and clinical manifestations of atherosclerosis has also come from the observation that cysteinyl-LTs induce contractions of human atherosclerotic coronary arteries, whereas nonatherosclerotic arteries are unresponsive[105]. Furthermore, with the identification of a distinctive expression pattern for the human $CysLT_2R$ in heart and vascular district, we have observed a renewed interest for cysteinyl-LT functions in the cardiovascular system. In human coronary artery, $LTC_4$-induced elevation of $[Ca^{2+}]_i$ was not blocked by $CysLT_1R$ classical antagonists, but blocked by the $Ca^{2+}$ channel blocker nicardipine, a known vascular relaxant[58]. Additionally, these cells showed chemotactic responses to $LTC_4$[58]. Taken together, these results strongly suggest that the activation of $CysLT_2R$ can induce profound effects in cardiac as well as in hemodynamic and microcirculatory pathophysiology, and that this receptor subtype will definitely represent an interesting pharmacological target for the future.

A recent study postulates that in a model of oxygen glucose deprivation (OGD)-induced cell death in PC12 cells, $CysLT_2R$ stimulation may facilitate the cell death, an event that was inhibited by BAY u9773, whereas $CysLT_1R$ reduces it[106].

### RECENT DISCOVERIES EXPANDING THE REPERTOIRE OF CYSLTR ACTIVITY/FUNCTIONS

Despite the classic view that the activity of cysteinyl-LTs is mainly due to the interaction with their two specific plasma membrane receptors, alternative pathways have been postulated, including localization of CysLTR at nuclear level, cross-talk with other membrane receptors, the possibility that CysLTR might exist as homo/heterodimers, and the existence of additional CysLTR subtypes (see the specific paragraphs below). All these experimental observations suggest major unanticipated roles for the cysteinyl-LT/CysLTR system in cellular signaling and function.

#### Signaling at the Nucleus

The intracrine CysLTR-induced signaling was first postulated in eosinophils[107], where $LTC_4$ was found to be tenfold more potent than $LTD_4$ in eliciting IL-4 release. This response was PTX sensitive, but more importantly insensitive to MK571 and BAY u9773, providing pharmacological evidence that the intracrine signaling of $LTC_4$ should be mediated by neither of the two known CysLTRs. Furthermore, $CysLT_1R$ was found to be located in the outer nuclear membrane in colon cancer cells or to be translocated into the nucleus after prolonged exposure to the agonist in nontransformed intestinal epithelial cells[108]. A nuclear localization sequence localized in the C-tail of the $CysLT_1R$ is essential for the targeting to the nucleus, as already demonstrated for other GPCRs[109,110], or proteins that normally localize to the nucleus[111].

It is tempting to speculate that nuclear CysLTR may exert peculiar and yet undiscovered roles as the existence of a functional intracellular GPCR population has already been observed for other GPCRs[112], which are also known to modulate gene expression of specific proinflammatory genes[113].

#### Cross-Talk with Other GPCRs

The activation of a number of receptors does not always lead to a direct effect on a particular signaling pathway, but rather to amplification or inhibition of the response produced by separate coincident signals within the same cell or tissue. It is now widely accepted that stimulation of particular GPCRs results in activation of signaling pathways that can subsequently interact with those activated by other receptors. This increase in complexity of signaling is expected considering cell diversity and the adjustments they have to exert in order to adapt to changes that take place during health and disease[114].

For example, cysteinyl-LTs may be postulated to play a role in the origin of the $\beta_2AR$ dysfunction caused by antigen challenge in sensitized human bronchi[115], because the dysfunction can be prevented by pretreatment with a $CysLT_1R$ antagonist[116]. Thus, it is possible that the presence of high levels of cysteinyl-LTs in the airways of asthmatic patients might desensitize the $\beta_2AR$[98], thus reducing the clinical efficacy of $\beta_2$ agonists. Accordingly, $LTD_4$ is indeed able to heterologously desensitize the $\beta_2AR$ in human airway SMCs and isolated human bronchi through the activation of PKC[97].

Recently, native human $CysLT_1R$ has been demonstrated to be the target for extracellular nucleotide-mediated heterologous desensitization[43]. Interestingly, ATP/UDP-induced $CysLT_1R$ desensitization, which was dependent on PKC, did not cause receptor internalization and induced a very fast recovery of

$CysLT_1R$ functionality with respect to $LTD_4$-induced homologous desensitization and trafficking[43]. Moreover, in the same system, $CysLT_1R$ antagonists inhibit the effects of nucleotides acting at P2Y receptors[117].

### Oligomerization

Some experimental evidence indicates the possibility that CysLTR might exist as homo- and/or heterodimers, starting from the observation of dimeric and oligomeric forms of $CysLT_1R$ in western blot and the punctate appearance of the immunohistochemical signal in PBLs[24] or U937 cells[70]. In human MCs, which can express both receptor subtypes, Mellor and colleagues observed that under conditions where $CysLT_1R$ is blocked, IL-5 generation results only from stimulation with BAY u9773 and not with cysteinyl-LTs[57], leading the authors to speculate that this could arise from stimulation of a $CysLT_1/CysLT_2$ heterodimer at a site inaccessible to interference from MK571. These speculations have been recently confirmed by assessing the formation of a $CysLT_1/CysLT_2$ heterodimer at the nuclear envelope of human MCs (J.A. Boyce, personal communication). Finally, Beller and colleagues[118], based on the magnitude of the attenuation in IgE-dependent, MC-mediated passive cutaneous anaphylaxis (PCA) in $CysLT_1R$ and $CysLT_2R$ null mice, postulated that the effect observed in wild-type littermates was mediated through $CysLT_1/CysLT_2$ heterodimers.

Though these observations are of potential interest considering that GPCR oligomerization offers new horizons to study important aspects of GPCR biology and possibly to develop new drugs[119,120], it has yet to be established if CysLTRs form homo/heterodimers. If so, how this influences their pharmacology and function, or is of any importance in cell physiology, is an issue that will clearly need to be addressed.

### CysLTR: Additional Subtypes

Over the years, several data were reported in the literature suggesting the existence of additional CysLTR subtypes in human tissues[5,121,122]. This proposal was based on the observation that one ligand ($LTE_4$[71,123,124] or $LTC_4$[76,125]) failed to activate a CysLTR or that the dual antagonist BAY u9773 failed to antagonize all cysteinyl-LT functional responses[124,126]. Furthermore, some authors have reported $LTC_4$ to be more potent than $LTD_4$ in inducing specific cellular functions[104,107], which, in the light of the known pharmacology from cloned receptors, does not fit with either $CysLT_1R$ or $CysLT_2R$ profile (see also below). These studies are supported by the results obtained from ligand binding studies that indicate the existence of a specific $LTC_4$ binding site in human lung parenchyma distinct from that of $LTD_4$[34], and which is not coupled to contraction[127]. Further support comes from the observation that in the same tissue, the most advanced $CysLT_1R$ antagonists display a different behavior toward $LTC_4$ and $LTD_4$[128]. In a report demonstrating that cysteinyl-LTs induced contractions of human atherosclerotic coronary arteries, Allen and colleagues[105] also suggest the involvement of a LT binding site specific for $LTC_4$.

In the light of the recent progress on the GPCR heterodimer pharmacology and their possible physiological significance, it is possible that these additional CysLTR subtypes might be the result of the formation of heterodimers with a different pharmacological profile, rather than representing new distinct proteins.

## DEORPHANIZATION OF A NEW DUAL CYSLT/UDP RECEPTOR

In 2001, in an intriguing report, Mellor and colleagues[53] suggested that both the $CysLT_1R$ and a yet-unidentified elusive receptor up-regulated by treatment with the proinflammatory cytokine IL-4 were responsive to both cysteinyl-LTs and UDP. Pharmacological studies performed with classical $CysLT_1R$ or the dual $CysLT_1$/$CysLT_2R$ antagonists excluded the possibility that this additional receptor was the $CysLT_2R$ subtype[53]. Very recently, we tested this hypothesis and screening for orphan GPCRs at an intermediate phylogenetic position between P2Y and CysLT receptor families, and found that the heterologous expression of GPR17 in different cell lines results in the appearance of highly specific and concentration-dependent responses to both cysteinyl-LTs and extracellular nucleotides[129]. Phylogenetically, GPR17 is equally distant from the $P2Y_{12,13,14}$ subgroup, and the $CysLT_1$ and $CysLT_2$ group[20], and consists of an open reading frame of 339 aa, as previously reported[130]. The basic characteristics of the human GPR17 receptor are summarized in Table 1.

### Pharmacological Characterization and Signal Transduction

[$^{35}$S]GTPγS binding assay of human GPR17 receptor transiently transfected in 1321N1 cells showed that $LTC_4$ was more potent ($EC_{50}$ = 0.33 n*M*) than $LTD_4$ ($EC_{50}$ = 7.2 n*M*). In the same cells, hGPR17 expression induced the appearance of concentration-dependent responses to UDP, UDP-glucose, and UDP-galactose with the rank order of potency UDP-galactose = UDP > UDP-glucose typically in the micromolar range. Thus, the agonist response profile of GPR17 is different from that of $CysLT_1$ and $CysLT_2$ receptors[7], and, for nucleotides, is intermediate between $P2Y_6$ and $P2Y_{14}$ receptors[131]. Both the cysteinyl-LT and nucleotide [$^{35}$S]GTPγS binding response were PTX sensitive, suggesting a functional coupling with a $G_{i/o}$-protein. In line with these results in 1321N1 cells expressing hGPR17, both cysteinyl-LTs and nucleotides show inhibition of forskolin-induced cAMP formation. Furthermore, in the same cell line, both agonists were also able to induce an increase in cytosolic $[Ca^{2+}]_i$.

The $LTD_4$ functional response is potently inhibited by the selective $CysLT_1R$ antagonists montelukast and pranlukast, with the latter sixfold more potent than the former. Moreover, cangrelor (formerly AR-C69931MX), a $P2Y_{12}$/$P2Y_{13}$ antagonist, and the $P2Y_1$-receptor antagonist MRS2179 concentration-dependently inhibited [$^{35}$S]GTPγS binding in cells expressing hGPR17, with $IC_{50}$ in the nanomolar range.

### Distribution and Functional Significance

In line with previous expression data[132], both human and rat GPR17 were highly present in brain and in other organs typically undergoing ischemic damage, such as kidney and heart, and with very low expression in liver and lung. These data are consistent with the demonstration that inhibition of GPR17 either by receptor antagonists (both $CysLT_1R$ and P2Y receptors) or *in vivo* receptor knock-down protects against brain damage in an established model of ischemic damage (the permanent monolateral middle cerebral artery occlusion in the rat[129]).

These data also add complexity to the already-established “cross-talk” between the purinergic and the LT receptor systems (see above), suggesting GPR17 as an additional means by which these two signaling systems interact with each other. It might be worth mentioning here that Nonaka and coworkers also reported that $LTE_4$ acted as an agonist at the P2Y12 receptor[133], adding another fragment to a picture that is becoming more and more complicated, yet more and more intriguing. Whether GPR17 is really a dualistic receptor (a single protomer responding to two different classes of ligands) or this new “pharmacological entity” is, in fact, a heterodimer between two distinct heptahelical proteins is a matter deserving further investigation.

### CONCLUSIONS

Cysteinyl-LTs exert a range of proinflammatory effects, such as constriction of airways and vascular smooth muscle, increase of endothelial cell permeability, induction of eosinophils chemotaxis, and enhanced mucus secretion. They have proved to be important mediators in asthma, allergic rhinitis, and other inflammatory conditions, including cardiovascular diseases, cancer, atopic dermatitis, and urticaria. With the cloning of $CysLT_1R$ and $CysLT_2R$, it has been confirmed that both were members of the GPCR superfamily, and that their localization, despite some overlapping, was peculiar. Recombinant CysLTRs couple to the $G_{q/11}$ pathway modulating IP hydrolysis and $Ca^{2+}$ mobilization, whereas in native systems, they also activate a PTX-sensitive $G_{i/o}$-protein, or are promiscuously coupled to both G-proteins. In constitutive human systems, $CysLT_1R$ increases $[Ca^{2+}]_i$ and PI metabolism, activates MAPKs, induces cell proliferation and differentiation, actin reorganization, chemotactic migration, release of various inflammatory mediators, and regulation of hematopoietic stem cells mobilization. $CysLT_2R$, besides increasing $[Ca^{2+}]_i$, stimulates IL-8 secretion; up-regulates early inducible genes; may be relevant to inflammation, hemostasis, thrombosis, and vascular injury; and facilitates cell death. Furthermore, alternative pathways have been postulated, including localization of CysLTR at nuclear level, cross-talk with other membrane receptors (EGF-R, $\beta_2AR$, P2Y-R), and the possibility that CysLTRs might exist as homo/heterodimers. These data seem to suggest major unanticipated roles for the cysteinyl-LT/CysLTR system in cellular signaling and function. Interestingly, recent data provide evidence for the existence of an additional receptor subtype that seems to respond to both cysteinyl-LTs and uracil nucleosides. GPR17 seems to be functionally coupled to a $G_{i/o}$-protein and to be implicated in ischemic damage.

In light of the recent progress on the GPCR heterodimer pharmacology and their possible physiological significance, it is possible that postulated additional CysLTR subtypes might be the result of the formation of heterodimers, rather than represent new distinct protein entities. If this is the case, a completely new array of physiological implications and, thus, of possible therapeutic interventions, is foreseen in the near future.

## References

1. Samuelsson, B. (1983) Leukotrienes: mediators of immediate hypersensitivity reactions and inflammation. *Science* **220,** 568–575.
2. Funk, C.D. (2001) Prostaglandins and leukotrienes: advances in eicosanoid biology. *Science* **294,** 1871–1875.
3. Capra, V., Thompson, M.D., Sala, A., Cole, D.E., Folco, G., and Rovati, G.E. (2006) Cysteinyl-leukotrienes and their receptors in asthma and other inflammatory diseases: critical update and emerging trends. *Med. Res. Rev.* **27(4),** 469–527.
4. Walch, L., Norel, X., Gascard, J.P., and Brink, C. (2000) Functional studies of leukotriene receptors in vascular tissues. *Am. J. Respir. Crit. Care Med.* **161,** S107–111.
5. Back, M. (2002) Functional characteristics of cysteinyl-leukotriene receptor subtypes. *Life Sci.* **71,** 611–622.
6. Evans, J.F. (2003) The cysteinyl leukotriene receptors. *Prostaglandins Leukot. Essent. Fatty Acids* **69,** 117–122.
7. Brink, C., Dahlen, S.E., Drazen, J., Evans, J.F., Hay, D.W., Nicosia, S., Serhan, C.N., Shimizu, T., and Yokomizo, T. (2003) International Union of Pharmacology XXXVII. Nomenclature for leukotriene and lipoxin receptors. *Pharmacol. Rev.* **55,** 195–227.
8. Capra, V. (2004) Molecular and functional aspects of human cysteinyl leukotriene receptors. *Pharmacol. Res.* **50,** 1–11.
9. Brooks, C.D. and Summers, J.B. (1996) Modulators of leukotriene biosynthesis and receptor activation. *J. Med. Chem.* **39,** 2629–2654.
10. Drazen, J.M., Austen, K.F., Lewis, R.A., Clark, D.A., Goto, G., Marfat, A., and Corey, E.J. (1980) Comparative airway and vascular activities of leukotrienes C-1 and D in vivo and in vitro. *Proc. Natl. Acad. Sci. U. S. A.* **77,** 4354–4358.
11. Fleisch, J.H., Rinkema, L.E., and Baker, S.R. (1982) Evidence for multiple leukotriene D4 receptors in smooth muscle. *Life Sci.* **31,** 577–581.
12. Krell, R.D., Tsai, B.S., Berdoulay, A., Barone, M., and Giles, R.E. (1983) Heterogeneity of leukotriene receptors in guinea-pig trachea. *Prostaglandins* **25,** 171–178.
13. Labat, C., Ortiz, J.L., Norel, X., Gorenne, I., Verley, J., Abram, T.S., Cuthbert, N.J., Tudhope, S.R., Norman, P., Gardiner, P., et al. (1992) A second cysteinyl leukotriene receptor in human lung. *J. Pharmacol. Exp. Ther.* **263,** 800–805.
14. Tudhope, S.R., Cuthbert, N.J., Abram, T.S., Jennings, M.A., Maxey, R.J., Thompson, A.M., Norman, P., and Gardiner, P.J. (1994) BAY u9773, a novel antagonist of cysteinyl-leukotrienes with activity against two receptor subtypes. *Eur. J. Pharmacol.* **264,** 317–323.
15. Lynch, K.R., Gary P. O'Neill, G.P., Qingyun Liu, Q., Im, D.-S., Sawyer, N., Metters, K.M., Coulombe, N., Abramovitz, M., Figueroa, D.J., Zeng, Z., Connolly, B.M., Bai, C., Austin, C.P., Chateauneuf, A., Stocco, R., Greig,

G.M., Kargman, S., Hooks, S.B., Hosfield, E., Williams Jr., D.L., Ford-Hutchinson, A.W., Caskey, C.T., and Evans, J.F. (1999) Characterization of the human cysteinyl leukotriene $CysLT_1$ receptor. *Nature* **399,** 789–793.

16. Sarau, H.M., Ames, R.S., Chambers, J., Ellis, C., Elshourbagy, N., Foley, J.J., Schmidt, D.B., Muccitelli, R.M., Jenkins, O., Murdock, P.R., Herrity, N.C., Halsey, W., Sathe, G., Muir, A.I., Nuthulaganti, P., Dytko, G.M., Buckley, P.T., Wilson, S., Bergsma, D.J., and Hay, D.W. (1999) Identification, molecular cloning, expression, and characterization of a cysteinyl leukotriene receptor. *Mol. Pharm*. **56,** 657–663.
17. Heise, C.E., O'Dowd, B.F., Figueroa, D.J., Sawyer, N., Nguyen, T., Im, D.-S., Stocco, R., Bellefeuille, J.N., Abramovitz, M., Cheng, R., Williams, D.L., Jr., Zeng, Z., Liu, Q., Ma, L., Clements, M.K., Coulombe, N., Liu, Y., Austin, C.P., George, S.R., O'Neill, G.P., Metters, K.M., Lynch, K.R., and Evans, J.F. (2000) Characterization of the human cysteinyl leukotriene 2 receptor. *J. Biol. Chem*. **275,** 30531–30536.
18. Takasaki, J., Kamohara, M., Matsumoto, M., Saito, T., Sugimoto, T., Ohishi, T., Ishii, H., Ota, T., Nishikawa, T., Kawai, Y., Masuho, Y., Isogai, T., Suzuki, Y., Sugano, S., and Furuichi, K. (2000) The molecular characterization and tissue distribution of the human cysteinyl leukotriene CysLT(2) receptor. *Biochem. Biophys. Res. Commun.* **274,** 316–322.
19. Nothacker, H.-P., Wang, Z., Zhu, Y., Reinscheid, R.K., Lin, S.H.S., and Civelli, O. (2000) Molecular cloning and characterization of a second human cysteinyl leukotriene receptor: discovery of a subtype selective agonist. *Mol. Pharmacol.* **58,** 1601–1608.
20. Fredriksson, R., Lagerstrom, M.C., Lundin, L.G., and Schioth, H.B. (2003) The G-protein-coupled receptors in the human genome form five main families. Phylogenetic analysis, paralogon groups, and fingerprints. *Mol. Pharmacol.* **63,** 1256–1272.
21. Kroeze, W.K., Sheffler, D.J., and Roth, B.L. (2003) G-protein-coupled receptors at a glance. *J. Cell Sci.* **116,** 4867–4869.
22. C. elegans Sequencing Consortium (1998) Genome sequence of the nematode C. elegans: a platform for investigating biology. *Science* **282**, 2012–2018.
23. Adams, M.D. et al. (2000) The genome sequence of Drosophila melanogaster. *Science* **287,** 2185–2195.
24. Figueroa, D.J., Breyer, R.M., Defoe, S.K., Kargman, S., Daugherty, B.L., Waldburger, K., Liu, Q., Clements, M., Zeng, Z., O'Neill, G.P., Jones, T.R., Lynch, K.R., Austin, C.P., and Evans, J.F. (2001) Expression of the cysteinyl leukotriene 1 receptor in normal human lung and peripheral blood leukocytes. *Am. J. Respir. Crit. Care Med.* **163,** 226–233.
25. Bautz, F., Denzlinger, C., Kanz, L., and Mohle, R. (2001) Chemotaxis and transendothelial migration of CD34(+) hematopoietic progenitor cells induced by the inflammatory mediator leukotriene D4 are mediated by the 7-transmembrane receptor CysLT1. *Blood* **97,** 3433–3440.
26. Sjostrom, M., Jakobsson, P.J., Heimburger, M., Palmblad, J., and Haeggstrom, J.Z. (2001) Human umbilical vein endothelial cells generate leukotriene C4 via microsomal glutathione S-transferase type 2 and express the CysLT(1) receptor. *Eur. J. Biochem*. **268,** 2578–2586.
27. Sjostrom, M., Jakobsson, P.J., Juremalm, M., Ahmed, A., Nilsson, G., Macchia, L., and Haeggstrom, J.Z. (2002) Human mast cells express two leukotriene C(4) synthase isoenzymes and the CysLT(1) receptor. *Biochim. Biophys. Acta* **1583,** 53–62.
28. Ohd, J.F., Nielsen, C.K., Campbell, J., Landberg, G., Lofberg, H., and Sjolander, A. (2003) Expression of the leukotriene D4 receptor CysLT1, COX-2, and other cell survival factors in colorectal adenocarcinomas. *Gastroenterology* **124,** 57–70.
29. Capra, V., Ravasi, S., Accomazzo, M.R., Parenti, M., and Rovati, G.E. (2004) CysLT1 signal transduction in differentiated U937 cells involves the activation of the small GTP-binding protein Ras. *Biochem. Pharmacol.* **67,** 1569–1577.
30. Woszczek, G., Pawliczak, R., Qi, H.Y., Nagineni, S., Alsaaty, S., Logun, C., and Shelhamer, J.H. (2005) Functional characterization of human cysteinyl leukotriene 1 receptor gene structure. *J. Immunol*. **175,** 5152–5159.
31. Zhang, J., Migita, O., Koga, M., Shibasaki, M., Arinami, T., and Noguchi, E. (2006) Determination of structure and transcriptional regulation of CYSLTR1 and an association study with asthma and rhinitis. *Pediatr. Allergy Immunol.* **17,** 242–249.
32. Lewis, M.A., Mong, S., Vessella, R.L., and Crooke, S.T. (1985) Identification and characterization of leukotriene $D_4$ receptors in adult and fetal human lung. *Biochem. Pharmacol.* **34**, 4311–4317.
33. Frey, E.A., Nicholson, D.W., and Metters, K.M. (1993) Characterization of the leukotriene D4 receptor in dimethylsulphoxide-differentiated U937 cells: comparison with the leukotriene D4 receptor in human lung and guinea-pig lung. *Eur. J. Pharmacol*. **244,** 239–250.
34. Capra, V., Nicosia, S., Ragnini, D., Mezzetti, M., Keppler, D., and Rovati, G.E. (1998) Identification and characterization of two cysteinyl-leukotriene high affinity binding sites with receptor characteristics in human lung parenchyma. *Mol. Pharmacol.* **53,** 750–758.
35. Capra, V., Accomazzo, M.R., Ravasi, S., Parenti, M., Macchia, M., Nicosia, S., and Rovati, G.E. (2003) Involvement of prenylated proteins in calcium signaling induced by LTD4 in differentiated U937 cells. *Prostaglandins Other Lipid Mediat.* **71,** 235–251.
36. Saussy, D.L., Jr., Sarau, H.M., Foley, J.J., Mong, S., and Crooke, S.T. (1989) Mechanisms of leukotriene $E_4$ partial agonist activity at leukotriene $D_4$ receptors in differentiated U-937 cells. *J. Biol. Chem.* **264**, 19845–19855.

37. Mong, S., Miller, J., Wu, H.-L., and Crooke, S.T. (1988) Leukotriene $D_4$ receptor-mediated hydrolysis of phosphoinositide and mobilization of calcium in sheep tracheal smooth muscle cells. *J. Pharmacol. Exp. Ther.* **244,** 508–515.
38. Kenakin, T. (1997) Differences between natural and recombinant G protein-coupled receptor systems with varying receptor/G protein stoichiometry. *Trends Pharmacol. Sci.* **18,** 456–464.
39. Sjölander, A., Grönroos, E., Hammarström, S., and Andersson, T. (1990) Leukotriene $D_4$ and $E_4$ induce transmembrane signaling in human epithelial cells. *J. Biol. Chem.* **265,** 20976–20981.
40. Hoshino, M., Izumi, T., and Shimizu, T. (1998) Leukotriene D4 activates mitogen-activated protein kinase through a protein kinase Calpha-Raf-1-dependent pathway in human monocytic leukemia THP-1 cells. *J. Biol. Chem.* **273,** 4878–4882.
41. Ravasi, S., Citro, S., Viviani, B., Capra, V., and Rovati, G.E. (2006) CysLT1 receptor-induced human airway smooth muscle cells proliferation requires ROS generation, EGF receptor transactivation and ERK1/2 phosphorylation. *Respir. Res*. **7**, 42.
42. Naik, S., Billington, C.K., Pascual, R.M., Deshpande, D.A., Stefano, F.P., Kohout, T.A., Eckman, D.M., Benovic, J.L., and Penn, R.B. (2005) Regulation of cysteinyl leukotriene type 1 receptor internalization and signaling. *J. Biol. Chem*. **280,** 8722–8732.
43. Capra, V., Ravasi, S., Accomazzo, M.R., Citro, S., Grimoldi, M., Abbracchio, M.P., and Rovati, G.E. (2005) CysLT1 receptor is a target for extracellular nucleotide-induced heterologous desensitization: a possible feedback mechanism in inflammation. *J. Cell Sci.* **118,** 5625–5636.
44. Drazen, J.M., Israel, E., and O'Byrne, P.M. (1999) Treatment of asthma with drugs modifying the leukotriene pathway. *N. Engl. J. Med.* **340**, 197–206.
45. Nicosia, S., Capra, V., and Rovati, G.E. (2001) Leukotrienes as mediators of asthma. *Pulm. Pharmacol. Ther.* **14,** 3–19.
46. Holgate, S.T., Peters-Golden, M., Panettieri, R.A., and Henderson, W.R., Jr. (2003) Roles of cysteinyl leukotrienes in airway inflammation, smooth muscle function, and remodeling. *J. Allergy Clin. Immunol.* **111,** S18–S36.
47. Sampson, A.P., Pizzichini, E., and Bisgaard, H. (2003) Effects of cysteinyl leukotrienes and leukotriene receptor antagonists on markers of inflammation. *J. Allergy Clin. Immunol.* **111,** S49–59; discussion S59–61.
48. Shirasaki, H., Kanaizumi, E., Watanabe, K., Matsui, T., Sato, J., Narita, S., Rautiainen, M., and Himi, T. (2002) Expression and localization of the cysteinyl leukotriene 1 receptor in human nasal mucosa. *Clin. Exp. Allergy* **32**, 1007–1012.
49. Figueroa, D.J., Borish, L., Baramki, D., Philip, G., Austin, C.P., and Evans, J.F. (2003) Expression of cysteinyl leukotriene synthetic and signalling proteins in inflammatory cells in active seasonal allergic rhinitis. *Clin. Exp. Allergy* **33**, 1380–1388.
50. Corrigan, C., Mallett, K., Ying, S., Roberts, D., Parikh, A., Scadding, G., and Lee, T. (2005) Expression of the cysteinyl leukotriene receptors cysLT(1) and cysLT(2) in aspirin-sensitive and aspirin-tolerant chronic rhinosinusitis. *J. Allergy Clin. Immunol.* **115,** 316–322.
51. Gauvreau, G.M., Plitt, J.R., Baatjes, A., and MacGlashan, D.W. (2005) Expression of functional cysteinyl leukotriene receptors by human basophils. *J. Allergy Clin. Immunol.* **116**, 80–87.
52. Gauvreau, G.M., Parameswaran, K.N., Watson, R.M., and O'Byrne, P.M. (2001) Inhaled leukotriene E(4), but not leukotriene D(4), increased airway inflammatory cells in subjects with atopic asthma. *Am. J. Respir. Crit. Care Med.* **164,** 1495–1500.
53. Mellor, E.A., Maekawa, A., Austen, K.F., and Boyce, J.A. (2001) Cysteinyl leukotriene receptor 1 is also a pyrimidinergic receptor and is expressed by human mast cells. *Proc. Natl. Acad. Sci. U. S. A.* **98,** 7964–7969.
54. Zhu, J., Qiu, Y.S., Figueroa, D.J., Bandi, V., Galczenski, H., Hamada, K., Guntupalli, K.K., Evans, J.F., and Jeffery, P.K. (2005) Localization and up-regulation of cysteinyl leukotriene-1 receptor in asthmatic bronchial mucosa. *Am. J. Respir. Cell Mol. Biol.* **33,** 531–540.
55. Mechiche, H., Candenas, L., Pinto, F.M., Nazeyrollas, P., Clement, C., and Devillier, P. (2004) Characterization of cysteinyl leukotriene receptors on human saphenous veins: antagonist activity of montelukast and its metabolites. *J. Cardiovasc. Pharmacol*. **43,** 113–120.
56. Zhang, W.P., Hu, H., Zhang, L., Ding, W., Yao, H.T., Chen, K.D., Sheng, W.W., Chen, Z., and Wei, E.Q. (2004) Expression of cysteinyl leukotriene receptor 1 in human traumatic brain injury and brain tumors. *Neurosci. Lett.* **363,** 247–251.
57. Mellor, E.A., Frank, N., Soler, D., Hodge, M.R., Lora, J.M., Austen, K.F., and Boyce, J.A. (2003) Expression of the type 2 receptor for cysteinyl leukotrienes (CysLT2R) by human mast cells: functional distinction from CysLT1R. *Proc. Natl. Acad. Sci. U. S. A.* **100(20),** 11589–11593.
58. Kamohara, M., Takasaki, J., Matsumoto, M., Matsumoto, S., Saito, T., Soga, T., Matsushime, H., and Furuichi, K. (2001) Functional characterization of cysteinyl leukotriene CysLT(2) receptor on human coronary artery smooth muscle cells. *Biochem. Biophys. Res. Commun.* **287,** 1088–1092.
59. Mita, H., Hasegawa, M., Saito, H., and Akiyama, K. (2001) Levels of cysteinyl leukotriene receptor mRNA in human peripheral leucocytes: significantly higher expression of cysteinyl leukotriene receptor 2 mRNA in eosinophils. *Clin. Exp. Allergy* **31,** 1714–1723.
60. Sjostrom, M., Johansson, A.S., Schroder, O., Qiu, H., Palmblad, J., and Haeggstrom, J.Z. (2003) Dominant expression of the CysLT2 receptor accounts for calcium signaling by cysteinyl leukotrienes in human umbilical vein endothelial cells. *Arterioscler. Thromb. Vasc. Biol.* **23,** E37–41.

61. Lotzer, K., Spanbroek, R., Hildner, M., Urbach, A., Heller, R., Bretschneider, E., Galczenski, H., Evans, J.F., and Habenicht, A.J. (2003) Differential leukotriene receptor expression and calcium responses in endothelial cells and macrophages indicate 5-lipoxygenase-dependent circuits of inflammation and atherogenesis. *Arterioscler. Thromb. Vasc. Biol.* **23,** E32–36.
62. Hu, H., Chen, G., Zhang, J.M., Zhang, W.P., Zhang, L., Ge, Q.F., Yao, H.T., Ding, W., Chen, Z., and Wei, E.Q. (2005) Distribution of cysteinyl leukotriene receptor 2 in human traumatic brain injury and brain tumors. *Acta Pharmacol. Sin*. **26,** 685–690.
63. Sloniewsky, D.E., Ridge, K.M., Adir, Y., Fries, F.P., Briva, A., Sznajder, J.I., and Sporn, P.H. (2004) Leukotriene D4 activates alveolar epithelial Na,K-ATPase and increases alveolar fluid clearance. *Am. J. Respir. Crit. Care Med.* **169,** 407–412.
64. Murphy, R.C., Hammaström, S., and Samuelsson, B. (1979) Leukotriene C: slow-reacting substance from murine mastocytoma cells. *Proc. Natl. Acad. Sci. U. S. A.* **76,** 4275–4279.
65. Nicholson, D.W., Ali, A., Klemba, M.W., Munday, N.A., Zamboni, R.J., and Ford-Hutchinson, A.W. (1992) Human leukotriene C4 synthase expression in dimethyl sulfoxide-differentiated U937 cells. *J. Biol. Chem.* **267,** 17849–17857.
66. Winkler, J.D., Sarau, H.M., Foley, J.J., and Crooke, S.T. (1988) Leukotriene D4-induced homologous desensitization in basal and differentiated U-937 cells: characterization with the partial agonist leukotriene E4 and assessment of receptor reserve. *J. Pharmacol. Exp. Ther*. **247**, 54–62.
67. Winkler, J.D., Sarau, H.M., Foley, J.J., and Crooke, S.T. (1988) Phorbol 12-myristate 13-acetate inhibition of leukotriene D4-induced signal transduction was rapidly reversed by staurosporine. *Biochem. Biophys. Res. Commun.* **157,** 521–529.
68. Pollock, K. and Creba, J. (1990) Leukotriene $D_4$ induced calcium changes in U937 cells may utilize mechanisms additional to inositol phosphate production that are pertussis toxin insensitive but are blocked by phorbol myristate acetate. *Cell. Signal.* **2,** 563–568.
69. Winkler, J.D., Sarau, H.M., Foley, J.J., and Crooke, S.T. (1990) Inhibitors of protein kinase C selectively enhanced leukotriene D4-induced calcium mobilization in differentiated U-937 cells. *Cell. Signal.* **2**, 427–437.
70. Capra, V., Ravasi, S., Accomazzo, M.R., Citro, S., Grimoldi, M., Abbracchio, M.P., and Rovati, G.E. (2005) CysLT1 receptor is a target for extracellular nucleotide-induced heterologous desensitization: a possible feedback mechanism in inflammation. *J. Cell Sci*. **118,** 5625–5636.
71. Chan, C.C., Ecclestone, P., Nicholson, D.W., Metters, K.M., Pon, D.J., and Rodger, I.W. (1994) Leukotriene D4-induced increases in cytosolic calcium in THP-1 cells: dependence on extracellular calcium and inhibition with selective leukotriene D4 receptor antagonists. *J. Pharmacol. Exp. Ther*. **269,** 891–896.
72. Dahlen, S.E., Hedqvist, P., Hammarström, S., and Samuelsson, B. (1980) Leukotrienes are potent costrictors of human bronchi. *Nature* **288,** 484–486.
73. Gorenne, I., Labat, C., Gascard, J.P., Norel, X., Nashashibi, N., and Brink, C. (1998) Leukotriene D4 contractions in human airways are blocked by SK&F 96365, an inhibitor of receptor-mediated calcium entry. *J. Pharmacol. Exp. Ther.* **284,** 549–552.
74. Snetkov, V.A., Hapgood, K.J., McVicker, C.G., Lee, T.H., and Ward, J.P. (2001) Mechanisms of leukotriene D4-induced constriction in human small bronchioles. *Br. J. Pharmacol.* **133,** 243–252.
75. Accomazzo, M.R., Rovati, G.E., Vigano, T., Hernandez, A., Bonazzi, A., Bolla, M., Fumagalli, F., Viappiani, S., Galbiati, E., Ravasi, S., Albertoni, C., Di Luca, M., Caputi, A., Zannini, P., Chiesa, G., Villa, A.M., Doglia, S.M., Folco, G., and Nicosia, S. (2001) Leukotriene D4-induced activation of smooth-muscle cells from human bronchi is partly Ca2+-independent. *Am. J. Respir. Crit. Care Med.* **163,** 266–272.
76. McMahon, B., Stenson, C., McPhillips, F., Fanning, A., Brady, H.R., and Godson, C. (2000) Lipoxin A4 antagonizes the mitogenic effects of leukotriene D4 in human renal mesangial cells. Differential activation of MAP kinases through distinct receptors. *J. Biol. Chem*. **275,** 27566–27575.
77. Jiang, Y., Kanaoka, Y., Feng, C., Nocka, K., Rao, S., and Boyce, J.A. (2006) Cutting edge: interleukin 4-dependent mast cell proliferation requires autocrine/intracrine cysteinyl leukotriene-induced signaling. *J. Immunol.* **177,** 2755–2759.
78. Saegusa, S., Tsubone, H., and Kuwahara, M. (2001) Leukotriene D(4)-induced Rho-mediated actin reorganization in human bronchial smooth muscle cells. *Eur. J. Pharmacol.* **413,** 163–171.
79. Parameswaran, K., Cox, G., Radford, K., Janssen, L.J., Sehmi, R., and O'Byrne, P.M. (2002) Cysteinyl leukotrienes promote human airway smooth muscle migration. *Am. J. Respir. Crit. Care Med.* **166**, 738–742.
80. Snyder, D.S., Castro, R., and Desforges, J.F. (1989) Antiproliferative effects of lipoxygenase inhibitors on malignant human hematopoietic cell lines. *Exp. Hematol*. **17**, 6–9.
81. Leikauf, G.D., Claesson, H.E., Doupnik, C.A., Hybbinette, S., and Grafstrom, R.C. (1990) Cysteinyl leukotrienes enhance growth of human airway epithelial cells. *Am. J. Physiol.* **259,** L255–261.
82. Rajah, R., Nunn, S.E., Herrick, D.J., Grunstein, M.M., and Cohen, P. (1996) Leukotriene D4 induces MMP-1, which functions as an IGFBP protease in human airway smooth muscle cells. *Am. J. Physiol.* **271,** L1014–1022.
83. Panettieri, R.A., Tan, E.M., Ciocca, V., Luttmann, M.A., Leonard, T.B., and Hay, D.W. (1998) Effects of $LTD_4$ on human airway smooth muscle cell proliferation, matrix expression, and contraction in vitro: differential sensitivity to cysteinyl leukotriene receptor antagonists. *Am. J. Respir. Cell Mol. Biol.* **19,** 453–461.

84. Espinosa, K., Bosse, Y., Stankova, J., and Rola-Pleszczynski, M. (2003) CysLT1 receptor upregulation by TGF-beta and IL-13 is associated with bronchial smooth muscle cell proliferation in response to LTD4. *J. Allergy Clin. Immunol.* **111**, 1032–1040.
85. Kumasawa, F., Hashimoto, S., Onose, A., Jibiki, I., Mizumura, K., Matsumoto, K., Maruoka, S., Gon, Y., Kobayashi, T., Takahashi, N., Ichijo, H., and Horie, T. (2005) Apoptosis signal-regulating kinase 1 in leukotriene D(4)-induced activator protein-1 activation in airway smooth muscle cells. *Eur. J. Pharmacol.* **517,** 11–16.
86. McMahon, B., Mitchell, D., Shattock, R., Martin, F., Brady, H.R., and Godson, C. (2002) Lipoxin, leukotriene, and PDGF receptors cross-talk to regulate mesangial cell proliferation. *FASEB J.* **16,** 1817–1819.
87. Braccioni, F., Dorman, S.C., O'Byrne P, M., Inman, M.D., Denburg, J.A., Parameswaran, K., Baatjes, A.J., Foley, R., and Gauvreau, G.M. (2002) The effect of cysteinyl leukotrienes on growth of eosinophil progenitors from peripheral blood and bone marrow of atopic subjects. *J. Allergy Clin. Immunol.* **110,** 96–101.
88. Perng, D.W., Wu, Y.C., Chang, K.T., Wu, M.T., Chiou, Y.C., Su, K.C., Perng, R.P., and Lee, Y.C. (2006) Leukotriene C4 induces TGF-beta1 production in airway epithelium via p38 kinase pathway. *Am. J. Respir. Cell Mol. Biol.* **34,** 101–107.
89. Przylipiak, A., Hafner, J., Przylipiak, J., Runnebaum, B., Rabe, T., and Kohn, F.M. (1998) Influence of leukotrienes on in vitro growth of human mammary carcinoma cell line MCF-7. *Eur. J. Obstet. Gynecol. Reprod. Biol.* **77,** 61–65.
90. Mattern, M.R., Mong, S., Mong, S.M., Bartus, J.O., Sarau, H.M., Clark, M.A., Foley, J.J., and Crooke, S.T. (1990) Transient activation of topoisomerase I in leukotriene D4 signal transduction in human cells. *Biochem. J.* **265,** 101–107.
91. Allen, S.P., Chester, A.H., Piper, P.J., Sampson, A.P., Akl, E.S., and Yacoub, M.H. (1992) Effects of leukotrienes C4 and D4 on human isolated saphenous veins. *Br. J. Clin. Pharmacol.* **34,** 409–414.
92. Ortiz, J.L., Gorenne, I., Cortijo, J., Seller, A., Labat, C., Sarria, B., Abram, T.S., Gardiner, P.J., Morcillo, E., and Brink, C. (1995) Leukotriene receptors on human pulmonary vascular endothelium. *Br. J. Pharmacol*. **115,** 1382–1386.
93. Mellor, E.A., Austen, K.F., and Boyce, J.A. (2002) Cysteinyl leukotrienes and uridine diphosphate induce cytokine generation by human mast cells through an interleukin 4-regulated pathway that is inhibited by leukotriene receptor antagonists. *J. Exp. Med.* **195,** 583–592.
94. Ichiyama, T., Hasegawa, M., Ueno, Y., Makata, H., Matsubara, T., and Furukawa, S. (2005) Cysteinyl leukotrienes induce monocyte chemoattractant protein 1 in human monocytes/macrophages. *Clin. Exp. Allergy* **35,** 1214–1219.
95. Thompson, C., Cloutier, A., Bosse, Y., Thivierge, M., Le Gouill, C., Larivee, P., McDonald, P.P., Stankova, J., and Rola-Pleszczynski, M. (2006) CysLT1 receptor engagement induces AP-1- and NF-{kappa}B-dependent interleukin-8 expression. *Am. J. Respir. Cell Mol. Biol.* **35(6),** 697–704.
96. Mohle, R., Boehmler, A.M., Denzlinger, C., and Kanz, L. (2003) Nonpeptide mediators in the hematopoietic microenvironment. *Ann. N. Y. Acad. Sci.* **996,** 61–66.
97. Rovati, G.E., Baroffio, M., Citro, S., Brichetto, L., Ravasi, S., Milanese, M., Crimi, E., and Brusasco, V. (2006) Cysteinyl-leukotrienes in the regulation of beta2-adrenoceptor function: an in vitro model of asthma. *Respir. Res*. **7,** 103.
98. Benovic, J.L. (2002) Novel beta2-adrenergic receptor signaling pathways. *J. Allergy Clin. Immunol.* **110,** S229–235.
99. Coreno, A., Skowronski, M., West, E., El-Ekiaby, A., and McFadden, E.R., Jr. (2005) Bronchoprotective effects of single doses of salmeterol combined with montelukast in thermally induced bronchospasm. *Chest* **127,** 1572–1578.
100. Heimburger, M. and Palmblad, J.E. (1996) Effects of leukotriene C4 and D4, histamine and bradykinin on cytosolic calcium concentrations and adhesiveness of endothelial cells and neutrophils. *Clin. Exp. Immunol*. **103,** 454–460.
101. Uzonyi, B., Lotzer, K., Jahn, S., Kramer, C., Hildner, M., Bretschneider, E., Radke, D., Beer, M., Vollandt, R., Evans, J.F., Funk, C.D., and Habenicht, A.J.R. (2006) Cysteinyl leukotriene 2 receptor and protease-activated receptor 1 activate strongly correlated early genes in human endothelial cells. *Proc. Natl. Acad. Sci. U. S. A.* **103(16)**, 6326–6331.
102. McIntyre, T.M., Zimmerman, G.A., and Prescott, S.M. (1986) Leukotrienes C4 and D4 stimulate human endothelial cells to synthesize platelet-activating factor and bind neutrophils. *Proc. Natl. Acad. Sci. U. S. A.* **83**, 2204–2208.
103. Datta, Y.H., Romano, M., Jacobson, B.C., Golan, D.E., Serhan, C.N., and Ewenstein, B.M. (1995) Peptido-leukotrienes are potent agonists of von Willebrand factor secretion and P-selectin surface expression in human umbilical vein endothelial cells. *Circulation* **92,** 3304–3311.
104. Pedersen, K.E., Bochner, B.S., and Undem, B.J. (1997) Cysteinyl leukotrienes induce P-selectin expression in human endothelial cells via a non-CysLT1 receptor-mediated mechanism. *J. Pharmacol. Exp. Ther.* **281**, 655–662.
105. Allen, S., Dashwood, M., Morrison, K., and Yacoub, M. (1998) Differential leukotriene constrictor responses in human atherosclerotic coronary arteries. *Circulation* **97**, 2406–2413.
106. Qian, X.D., Wei, E.Q., Zhang, L., Sheng, W.W., Wang, M.L., Zhang, W.P., and Chen, Z. (2006) Pranlukast, a cysteinyl leukotriene receptor 1 antagonist, protects mice against brain cold injury. *Eur. J. Pharmacol.* **549,** 35–40.
107. Bandeira-Melo, C., Woods, L.J., Phoofolo, M., and Weller, P.F. (2002) Intracrine cysteinyl leukotriene receptor-mediated signaling of eosinophil vesicular transport-mediated interleukin-4 secretion. *J. Exp. Med.* **196,** 841–850.
108. Nielsen, C.K., Campbell, J.I., Ohd, J.F., Morgelin, M., Riesbeck, K., Landberg, G., and Sjolander, A. (2005) A novel localization of the G-protein-coupled CysLT1 receptor in the nucleus of colorectal adenocarcinoma cells. *Cancer Res.* **65,** 732–742.

109. Lu, D., Yang, H., Shaw, G., and Raizada, M.K. (1998) Angiotensin II-induced nuclear targeting of the angiotensin type 1 (AT1) receptor in brain neurons. *Endocrinology* **139,** 365–375.
110. Lee, D.K., Lanca, A.J., Cheng, R., Nguyen, T., Ji, X.D., Gobeil, F., Jr., Chemtob, S., George, S.R., and O'Dowd, B.F. (2004) Agonist-independent nuclear localization of the Apelin, angiotensin AT1, and bradykinin B2 receptors. *J. Biol. Chem*. **279,** 7901–7908.
111. Hanaka, H., Shimizu, T., and Izumi, T. (2002) Nuclear-localization-signal-dependent and nuclear-export-signal-dependent mechanisms determine the localization of 5-lipoxygenase. *Biochem. J*. **361**, 505–514.
112. Gobeil, F., Fortier, A., Zhu, T., Bossolasco, M., Leduc, M., Grandbois, M., Heveker, N., Bkaily, G., Chemtob, S., and Barbaz, D. (2006) G-protein-coupled receptors signalling at the cell nucleus: an emerging paradigm. *Can. J. Physiol. Pharmacol*. **84**, 287–297.
113. Marrache, A.M., Gobeil, F., Zhu, T., and Chemtob, S. (2005) Intracellular signaling of lipid mediators via cognate nuclear G protein-coupled receptors. *Endothelium* **12,** 63–72.
114. Selbie, L.A. and Hill, S.J. (1998) G protein-coupled-receptor cross-talk: the fine-tuning of multiple receptor-signalling pathways. *Trends Pharmacol. Sci*. **19**, 87–93.
115. Song, P., Milanese, M., Crimi, E., Rehder, K., and Brusasco, V. (1997) Allergen challenge of passively sensitized human bronchi alters M2 and beta2 receptor function. *Am. J. Respir. Crit. Care Med.* **155**, 1230–1234.
116. Song, P., Crimi, E., Milanese, M., Duan, J., Rehder, K., and Brusasco, V. (1998) Anti-inflammatory agents and allergen-induced beta2-receptor dysfunction in isolated human bronchi. *Am. J. Respir. Crit. Care Med.* **158,** 1809–1814.
117. Mamedova, L., Capra, V., Accomazzo, M.R., Gao, Z.G., Ferrario, S., Fumagalli, M., Abbracchio, M.P., Rovati, G.E., and Jacobson, K.A. (2005) CysLT(1) leukotriene receptor antagonists inhibit the effects of nucleotides acting at P2Y receptors. *Biochem. Pharmacol.* **71**, 115–125.
118. Beller, T.C., Maekawa, A., Friend, D.S., Austen, K.F., and Kanaoka, Y. (2004) Targeted gene disruption reveals the role of the cysteinyl leukotriene 2 receptor in increased vascular permeability and in bleomycin-induced pulmonary fibrosis in mice. *J. Biol. Chem*. **279,** 46129–46134.
119. Maggio, R., Novi, F., Scarselli, M., and Corsini, G.U. (2005) The impact of G-protein-coupled receptor hetero-oligomerization on function and pharmacology. *FEBS J.* **272**, 2939–2946.
120. Bulenger, S., Marullo, S., and Bouvier, M. (2005) Emerging role of homo- and heterodimerization in G-protein-coupled receptor biosynthesis and maturation. *Trends Pharmacol. Sci*. **26**, 131–137.
121. Rovati, G.E., Capra, V., and Nicosia, S. (1997) More on the classification of cysteinyl leukotriene receptors. *Trends Pharmacol. Sci.* **18,** 148.
122. Norel, X. and Brink, C. (2004) The quest for new cysteinyl-leukotriene and lipoxin receptors: recent clues. *Pharmacol. Ther.* **103**, 81–94.
123. Schellenberg, R.R. and Foster, A. (1984) Differential activity of leukotrienes upon human pulmonary vein and artery. *Prostaglandins* **27,** 475–482.
124. Walch, L., Norel, X., Back, M., Gascard, J.P., Dahlen, S.E., and Brink, C. (2002) Pharmacological evidence for a novel cysteinyl-leukotriene receptor subtype in human pulmonary artery smooth muscle. *Br. J. Pharmacol.* **137,** 1339–1345.
125. Ohd, J.F., Wikstrom, K., and Sjolander, A. (2000) Leukotrienes induce cell-survival signaling in intestinal epithelial cells. *Gastroenterology* **119,** 1007–1018.
126. Back, M., Norel, X., Walch, L., Gascard, J., de Montpreville, V., Dahlen, S., and Brink, C. (2000) Prostacyclin modulation of contractions of the human pulmonary artery by cysteinyl-leukotrienes. *Eur. J. Pharmacol.* **401,** 389–395.
127. Ravasi, S., Capra, V., Mezzetti, M., Nicosia, S., and Rovati, G.E. (2000) A kinetic binding study to evaluate the pharmacological profile of a specific leukotriene C(4) binding site not coupled to contraction in human lung parenchyma. *Mol. Pharmacol.* **57,** 1182–1189.
128. Ravasi, S., Capra, V., Panigalli, T., Rovati, G.E., and Nicosia, S. (2002) Pharmacological differences among CysLT(1) receptor antagonists with respect to LTC(4) and LTD(4) in human lung parenchyma. *Biochem. Pharmacol.* **63,** 1537–1546.
129. Ciana, P., Fumagalli, M., Trincavelli, M.L., Verderio, C., Rosa, P., Lecca, D., Ferrario, S., Parravicini, C., Capra, V., Gelosa, P., Guerrini, U., Belcredito, S., Cimino, M., Sironi, L., Tremoli, E., Rovati, G.E., Martini, C., and Abbracchio, M.P. (2006) The orphan receptor GPR17 identified as a new dual uracil nucleotides/cysteinyl-leukotrienes receptor. *EMBO J.* **25,** 4615–4627.
130. Raport, C.J., Schweickart, V.L., Chantry, D., Eddy, R.L., Jr., Shows, T.B., Godiska, R., and Gray, P.W. (1996) New members of the chemokine receptor gene family. *J. Leukoc. Biol.* **59,** 18–23.
131. Abbracchio, M.P., Burnstock, G., Boeynaems, J.M., Barnard, E.A., Boyer, J.L., Kennedy, C., Fumagalli, M., Gachet, C., Jacobson, K.A., and Weisman, G.A. (2006) International Union of Pharmacology LVIII: update of the P2Y G protein-coupled nucleotide receptors: from molecular mechanisms and pathophysiology to therapy. *Pharmacol. Rev.* **58,** 281–341.
132. Blasius, R., Weber, R.G., Lichter, P., and Ogilvie, A. (1998) A novel orphan G protein-coupled receptor primarily expressed in the brain is localized on human chromosomal band 2q21. *J. Neurochem*. **70,** 1357–1365.

133. Nonaka, Y., Hiramoto, T., and Fujita, N. (2005) Identification of endogenous surrogate ligands for human P2Y12 receptors by in silico and in vitro methods. *Biochem. Biophys. Res. Commun.* **337,** 281–288.

134. Massoumi, R. and Sjolander, A. (2007) The Role of Leukotriene Receptor Signaling in Inflammation and Cancer. *TheScientificWorldJOURNAL* **7**, 1413-1421.

135. Bäck, M. (2007) Leukotriene Receptors: Crucial Components in Vascular Inflammation. T*heScientificWorldJOURNAL* **7**, 1422-1439.
